# Improved protocol for the vitrification and warming of rat zygotes by optimizing the warming solution and oocyte donor age

**DOI:** 10.1371/journal.pone.0328718

**Published:** 2025-09-08

**Authors:** Naomi Nakagata, Satohiro Nakao, Nobuyuki Mikoda, Katsuma Yamaga, Yoshiko Nakagawa, Tetsushi Sakuma, Takashi Yamamoto, Toru Takeo

**Affiliations:** 1 Division of Reproductive Biotechnology and Innovation, Center for Animal Resources and Development, Institute of Resource Development and Analysis, Kumamoto University, Kumamoto, Japan; 2 Division of Reproductive Engineering, Center for Animal Resources and Development, Institute of Resource Development and Analysis, Kumamoto University, Kumamoto, Japan; 3 Kyudo Co., Ltd., Saga, Japan; 4 Laboratory of Genome-Editing Breeding, Graduate School of Agriculture, Kyoto University, Kyoto, Japan; 5 Genome Editing Innovation Center, Hiroshima University, Higashi‐Hiroshima, Japan; 6 Division of Integrated Sciences for Life, Graduate School of Integrated Sciences for Life, Hiroshima University, Higashi‐Hiroshima, Japan; University of Massachusetts Amherst, UNITED STATES OF AMERICA

## Abstract

Zygotes are used to create genetically modified animals by electroporation using the CRISPR-Cas9 system. Such zygotes in rats are obtained from superovulated female rats after mating. Recently, we reported that *in vivo-*fertilized zygotes had higher cryotolerance and developmental ability than *in vitro-*fertilized zygotes in Sprague Dawley (SD) and Fischer 344 rats. To apply the *in vitro*-fertilized zygotes in creating genetically modified rats, we need to address their low cryotolerance and developmental ability. Hence, we evaluated the effects of warming solutions containing different sucrose concentrations (0–0.3 M) and the oocyte donor’s age (3–7-week-old SD rats) on the viability of vitrified-warmed zygotes after *in vitro* fertilization and on developmental ability by embryo transfer in SD rats. A warming solution containing 0.1 M sucrose enhanced the survival rate of vitrified-warmed zygotes and their rate of development to two-cell embryos. Additionally, zygotes derived from 6- and 7-week-old female rats had higher cryotolerance and developmental ability than those from 3-week-old ones. Next, vitrified-warmed rat zygotes produced using the optimized protocol underwent genome editing by electroporation with Cas9 ribonucleoprotein and gRNA introduced to disrupt the *Tyr* gene. We then found that 86.5% of the pups derived from zygotes demonstrated mutation of the targeted gene. Therefore, the improved protocol for vitrifying and warming rat zygotes is useful for preserving and producing genetically modified rats.

## Introduction

Applying genome-editing technology to animals has advanced life sciences and technologies [1]. In the biomedical sciences, laboratory rats are widely used for physiological, pharmacological, toxicological, and behavioral analyses. Furthermore, genetically modified rats contribute to the understanding of human diseases and the evaluation of the efficacy and safety of drugs and medical applications in the biomedical industries [2]. Therefore, various genetically modified rats have been developed via genome-editing technology to date [3].

Zygotes are used to produce genetically modified rats using the genome-editing system with electroporation or microinjection [4]. Electroporation can be easily performed, simplifying the production of genetically modified rats by genome-editing technology. Using the cryopreservation of rat zygotes produced by mating also improved the production processes of genetically modified rats [5,6]. Recently, we found that the *in vivo*-fertilized zygotes have higher cryotolerance and are more suitable for cryopreservation than the *in vitro-*fertilized zygotes [7]. However, the lower success and fertilization rates of mating depending on rat strains limit the use of *in vivo*-fertilized zygotes.

*In vitro* fertilization (IVF) can efficiently produce zygotes. One study reported the efficient production of genetically modified rats using zygotes produced via IVF and a genome-editing system [8]. However, we previously reported that the low cryotolerance and developmental ability of *in vitro*-fertilized rat zygotes remain a technical problem [7,9].

To solve the problems of cryopreserving IVF-derived rat zygotes, this study aimed to examine the effects of warming solution at various sucrose concentrations (0, 0.05, 0.1, 0.2, or 0.3 M) or oocyte donors’ age (3, 4, 5, 6, or 7 weeks old) on the survival and developmental rate of vitrified-warmed zygotes produced by IVF. We also sought to evaluate whether these vitrified-warmed zygotes and electroporation can efficiently produce genetically modified rats (S1 Fig).

## Materials and methods

### Animals

Male (Crl:CD[SD] and Crlj:LE strain) and female (Crl:CD[SD] strain) rats were purchased from Jackson Laboratory Japan (Kanagawa, Japan). We utilized male rats aged 11–13 weeks (300–350 g body weight) as sperm donors, female rats aged 3–7 weeks (60–200 g body weight) as oocyte donors, and female rats aged 8–15 weeks (200–300 g body weight) as recipients for embryo transfer. All animals were housed in the laminar flow rack under a 12 h dark/light cycle (light from 7:00 to 19:00) at 22°C ± 2°C with *ad libitum* access to food (CE-2, CLEA Japan, Inc., Tokyo, Japan) and water. The rats were kept in cages, with three rats per cage. They all maintained healthy conditions, and none required early euthanasia according to the predefined human endpoints. The Institutional Animal Care and Use Committee of Kumamoto University approved the protocols for the animal experiments (A2023-079), and all experiments were conducted in the accordance with the ARRIVE guidelines and relevant guidelines and regulations.

### IVF

IVF was performed as previously described [9–12]. Females at 3–7 weeks old were injected with 0.2–0.3 mL of CARD HyperOva for Rat (KYD-FR-003, COSMOBIO, Tokyo, Japan). After 54–56 h, human chorionic gonadotropin (hCG, ASKA Animal Health Co. Ltd, Japan) was administered at a dose of 20 IU (3–5 weeks old) or 30 IU for (6–7 weeks old). For 6- to 7-week-old females, 0.04 mg (dissolved in 200 μL of saline) of (des-Gly10, D-Ala6)-LH-RH ethylamide acetate salt hydrate (L4513, Sigma-Aldrich) was administered at 48–50 h before injecting CARD HyperOva for Rat [8].

After euthanizing the male rats by cervical dislocation under inhalation anesthesia with isoflurane, we removed their cauda epididymides and placed them in paraffin oil. In each of the cauda epididymides, a short incision was made using micro-scissors, and the sperm were transferred into a 400 μL drop of modified human tubal fluid (mHTF) [13,14] using a glass rod (15 mm). The sperm suspension was incubated for 10 min at 37°C with 5% CO_2_. Sperm concentration was calculated using a hemocytometer (Erma, Tokyo, Japan) before the sperm suspensions were added to a drop of 200 μL of mHTF (fertilization medium) covered with paraffin oil (final concentration: 500 sperm/μL). Then, the sperm were incubated for 2 h at 37°C in a 5% CO_2_ environment to induce capacitation, followed by IVF.

At 15–16 h after hCG injection, we sacrificed the female rats via cervical dislocation and promptly collected their oviducts. All intact cumulus oocyte complexes were then released and transferred from the oviducts into the fertilization medium with sperm (insemination).

At 6–7 h after insemination, the oocytes and zygotes were observed under an inverted microscope (200×). The fertilization rates were calculated as the total number of zygotes (two pronuclei and one sperm tail) divided by the total number of inseminated oocytes multiplied by 100. Then, the zygotes were collected under a stereomicroscope (15×). The rates of fertilization via IVF were >90% in all experiments.

### Vitrification of rat zygotes

The procedures for zygotes vitrification were carried out as previously described [15]. The zygotes used in the experiments were randomly selected from the fertilization medium and allocated to each group before undergoing vitrification. First, 20–30 zygotes were pretreated with PB1 [16] containing 1 M of dimethyl sulfoxide (DMSO) at room temperature (25°C). Then, 5 μL of the solution, with the zygotes, was transferred into a 1.2 mL cryotube (Cat. No.: MS-4501W; Sumitomo Bakelite, Japan). We then placed the samples in a block cooler (Cat. No.: 5115−0012, Nalgene) at 0°C for 5 min. Then, 45 μL of a vitrification solution (DAP213; 2 M DMSO, 1 M acetamide, and 3 M propylene glycol in PB1) [17] was added at 0°C to each cryotube. After another 5 min, the samples were plunged directly into liquid nitrogen and stored for 3–10 weeks before warming. In each cryotube, we cryopreserved 20 zygotes in Experiment 1 and 30 zygotes in Experiments 2 and 3.

### Warming of the cryopreserved zygotes

The cryotubes containing the zygotes were collected from the liquid nitrogen, warmed at room temperature (25°C) for 60 s. Then, 0.9 mL of PB1 with several concentrations of sucrose (0, 0.05, 0.1, 0.2, or 0.3 M), prewarmed at 37°C, was added to these tubes. The contents of each tube were transferred to a plastic dish (Cat. No.: 430588, Corning) to recover the zygotes. Before the experiment, the osmotic pressure for each sucrose concentration was measured using an osmometer (Gonotec Osmomat 030-D, ELITechGroup Inc., France). The recovered zygotes were washed in three drops of PB1 (60 μL/drop) and placed into a 100 μL-drop of mHTF, and their morphological appearance was recorded (Experiment 1). Morphologically normal zygotes were defined as those with no damage to the zona pellucida/cytoplasm and no deformation of the cytoplasm (i.e., zygotes with the same morphology as before vitrification) [18]. In Experiment 2, the morphologically normal zygotes were transferred to the recipients approximately 30 min after warming.

### gRNA synthesis and Cas9 preparation

*In vitro*-transcribed gRNAs were prepared according to a previous report [19,20]. The gRNA designs of the *Tyr* gene were as previously described [21]. This *Tyr* target sequence existed in exon1 of the SD and LE rat genomes as a perfect match. Recombinant Cas9 protein was obtained from Integrated DNA Technologies in Japan (Alt-R™ S.p. Cas9 Nuclease 3NLS; Tokyo, Japan).

### Electroporation

Electroporation was performed according to a previous report [20,22]. After rinsing the zygotes with Opti-MEM I (Thermo Fisher Scientific), we placed them in the electrode gap filled with 5 µL of Opti-MEM I solution containing Cas9 protein and gRNA. Using an electrode (LF501PT1–10; BEX, Tokyo, Japan) and Genome Editor (GEB15, BEX), we performed electroporation seven times at 20 V (3 ms ON + 97 ms OFF). Thereafter, we rinsed the zygotes with M2 medium (Sigma, Tokyo, Japan) and cultured them in mHTF at 37°C in 5% CO_2_ and 95% humidified air until transfer.

### Transfer of vitrified-warmed zygotes into the recipients

In preparing female recipients, females at the proestrus observed by vaginal smears were mated with vasectomized male rats the day before the transfer procedures. The females with vaginal plug were used for transfer. A mixture of three anesthetic agents, medetomidine hydrochloride (0.375 mg/kg), midazolam (2 mg/kg), and butorphanol tartrate (2.5 mg/kg), was administered at a dose of 0.5 mL per 100 g of body weight. Afterward, 20–31 zygotes were transferred into the oviducts of a recipient [23]. The antagonist (Antisedan, 150 μg/mL) was administered at 0.5 mL/100 g of body weight and the recipients were kept on a hotplate maintained at 37°C until the recipients woke up. At 22–23 days after embryo transfer, we counted the number of live pups. The birth rate was calculated as the number of live pups divided by the number of transferred zygotes and multiplied by 100.

### Analysis of the pups

Pup tail lysates were prepared via an alkaline lysis method, and the polymerase chain reaction (PCR) for the heteroduplex mobility assay (HMA) was conducted using KOD FX (Toyobo, Osaka, Japan) and a primer set as reported previously [20]. For *Tyr* gene analysis, we confirmed the coat color of each pup and used the pup tail lysates for the PCR. Each PCR product of the *Tyr* gene underwent automatic electrophoresis using MultiNA (Shimadzu Corporation, Kyoto, Japan) for HMA analysis [24]. The PCR products indicating a single band of HMA were analyzed by direct sequencing using ABI 3130 Genetic Analyzer (Thermo Fisher Scientific) with BigDye Terminator v3.1 Cycle Sequencing Kit (Thermo Fisher Scientific). We then used PtWAVE, a Tracking of Insertions and Deletions analysis tool, for identifying mutations [25].

### Statistical analysis

Statistical data were analyzed using GraphPad Prism version 8.0 (GraphPad Software, Boston, MA, USA). The results are expressed as the mean ± standard deviation. Results were compared using Student’s *t*-test or, one-way analysis of variance (ANOVA) and Dunnett’s comparisons test, or Tukey’s multiple comparisons test, with *p* < 0.05 considered statistically significant.

## Results

### Experiment 1: 0.1 M sucrose solution enhanced the developmental rate of vitrified-warmed rat zygotes

Zygotes were produced via IVF between oocytes collected from 4-week-old Clr:CD females and sperm collected from males of the same strain rats. We vitrified and warmed 100 zygotes (20 zygotes/cryotube × 5) in each experimental group to examine the effect of the sucrose concentration used for warming.

**Fig 1:**
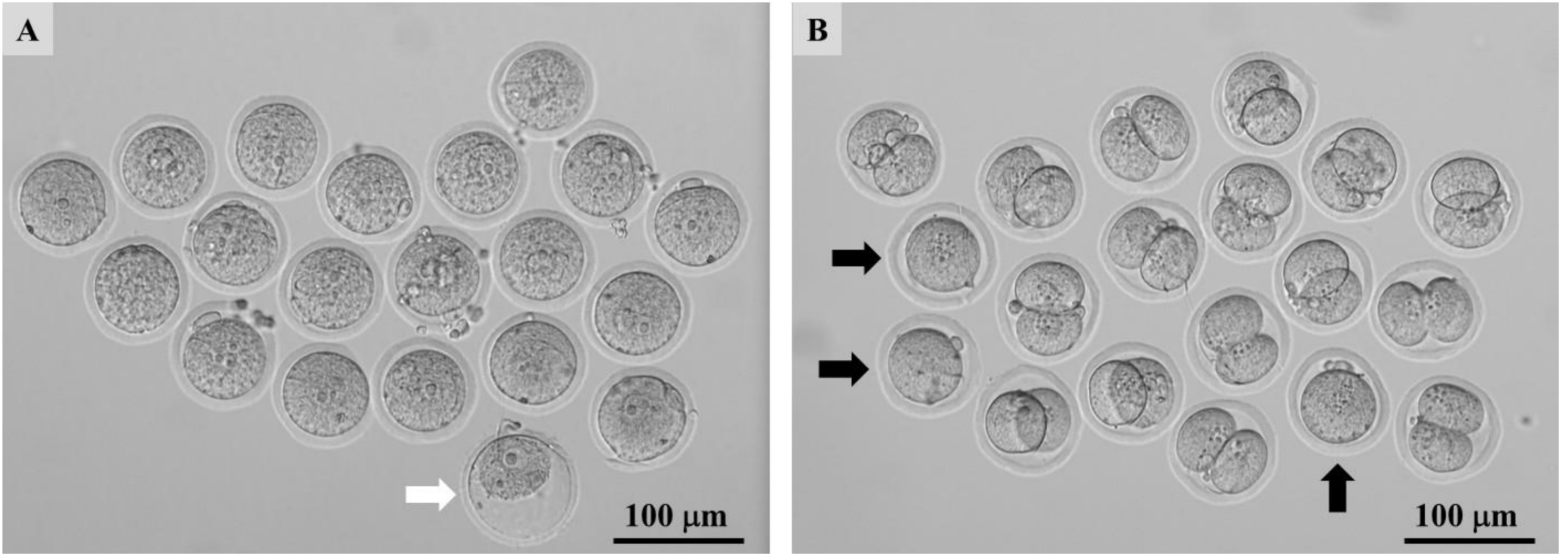
Vitrified-warmed zygotes derived from 7-week-old females. Vitrified-warmed zygotes derived from 7-week-old females (A). All zygotes were morphologically normal, except for one, which died (white arrow). Two-cell embryos derived from vitrified-warmed zygotes from 7-week-old females at 24 h after culture (B). While 3 out of 19 zygotes did not undergo cleavage (black arrow), the remaining 16 developed into two-cell embryos.

The vitrified–warmed rat zygotes exhibited cytoplasmic lysis and failed to develop into two-cell embryos. Therefore, we evaluated the recovery and survival rates of zygotes after vitrification and warming as well as the developmental rate of these recovered zygotes into two-cell embryos. The recovery and survival rates did not change in all groups (Table 1, Fig 1A, S1 Table). Sucrose solution at 0.1 M improved the rate of development into two-cell embryos after a 24 h culture of vitrified-warmed zygotes compared with 0 mM sucrose solution (*p* = 0.0122) (Table 1, Fig 1B, S1 Table). On the basis of these results, 0.1 M sucrose solution was used for warming in the following experiments.

**Table 1:** Effects of sucrose concentrations on the survival and developmental ability of vitrified-warmed rat zygotes.

Sucros (M)	Osmolality (mOsm)	No. of vitrified zygotes	No. of recovered zygotes (%)	No. of survived zygotes (%)	No. of two-cell embryos (%)
0	291	100	97 (97 ± 2.7)	63 (64.9 ± 23.9)	42 (43.3 ± 16.8)
0.05	347	100	98 (98 ± 2.7)	73 (74.5 ± 24.1)	55 (56.1 ± 21.5)
0.1	408	100	98 (98 ± 2.7)	91 (92.9 ± 6.8)	78 (79.6 ± 13.6)^*^
0.2	518	100	95 (95 ± 5.0)	69 (72.6 ± 21.4)	51 (53.7 ± 23.9)
0.3	646	100	98 (98 ± 2.7)	65 (66.3 ± 14.9)	37 (37.8 ± 6.5)

The recovery, developmental, and survival rates were calculated as follows: recovery rate = total number of recovered zygotes/total number of vitrified zygotes × 100, survival rate = total number of survived zygotes/total number of recovered zygotes × 100, and developmental rate into two-cell embryos = total number of two-cell embryos/total number of recovered zygotes × 100. The recovery rate, survival rate and developmental rate into two-cell embryos in each experimental group were compared with 0 mM sucrose using one-way ANOVA and Dunnett’s comparisons test (^*^p < 0.05, *n* = 5). In total, 30 female and male rats were used in this experiment.

### Experiment 2: Oocytes collected from 7-week-old female rats showed the highest developmental rates of vitrified-warmed zygotes

Zygotes were produced via IVF between oocytes collected from 3- to 7-week-old Clr:CD females and sperm collected from males of the same strain at 11–13 weeks old. In total, 65 female rats were used for IVF (10, 10, 10, 15 and 20 rats aged 3, 4, 5, 6, and 7 weeks, respectively). Vitrified zygotes derived from 3-, 4-, 5-, 6-, and 7-week-old females (150 zygotes per group, 30 zygotes/cryotube × 5) were warmed. The number of animals required to produce 150 zygotes was as follows: 6, 6, 6, 10, and 12 females aged 3, 4, 5, 6, and 7 weeks, respectively. Most of the vitrified zygotes were recovered after warming, and 79%–97% of them were morphologically normal in each group (Table 2, S2 Table). However, the vitrified zygotes from 3-week-old rats exhibited a lower survival rate than those from 4- to 7-week-old rats (Table 2, S2 Table) (*p* value vs 4-week-old: 0.0003, vs 5-week-old: 0.0003, vs 6-week-old: < 0.0001, and vs 7-week-old: < 0.0001).

**Table 2:** Effect of oocyte donors’ age on the survival rate of vitrified-warmed rat zygotes.

Age of the females (weeks)	No. of vitrified zygotes	No. of recovered zygotes (%)	No. of survived zygotes (%)
3	150	140 (93.3 ± 2.4)	110 (78.6 ± 4.7)^a^
4	150	139 (92.7 ± 1.5)	129 (92.8 ± 2.5)^b^
5	150	140 (93.3 ± 4.1)	130 (92.9 ± 6.7)^b^
6	150	146 (97.3 ± 2.8)	139 (95.2 ± 1.8)^b^
7	150	148 (98.7 ± 3.0)	144 (97.3 ± 3.7)^b^

The recovery rate was calculated by total number of recovered zygotes/total number of vitrified zygotes × 100. The survival rate was calculated by total number of survived zygotes/total number of recovered zygotes × 100. The recovery and survival rates were compared between all experimental groups using one-way ANOVA and Tukey’s multiple comparisons test. The different letters indicate significant differences (*p* < 0.05, *n* = 5).

**Fig 2:**
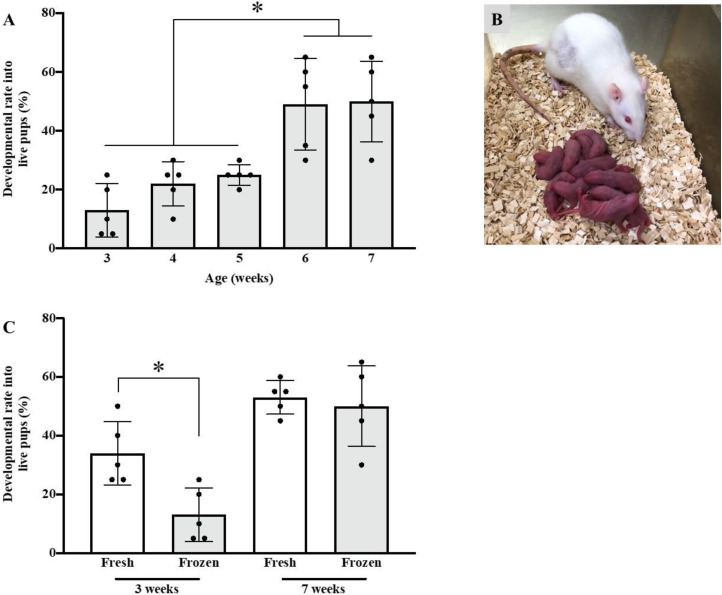
Developmental rate of vitrified-warmed zygotes from females at several weeks old. Zygotes derived from 3- to 7-week-old females were vitrified and warmed for embryo transfer. The developmental rate of live pups was evaluated (A). At 22–23 days after embryo transfer, live pups from vitrified-warmed zygotes derived from 3- to 7-week-old females were counted (B: from 7 weeks old). The rate of development into pups of fresh and vitrified-warmed zygotes derived from 3- and 7-week-old females was also evaluated (C). The results are expressed as mean ± standard deviation (*n* = 5). The developmental rates into pups were compared using one-way ANOVA and Tukey’s multiple comparisons test between all vitrified-warmed groups (A) or Student’s *t*-test between Fresh and Frozen in same age (B) (*p < 0.05).

To evaluate the developmental ability of the vitrified-warmed zygotes obtained from 3- to 7-week-old females, we used 100 of the recovered morphologically normal zygotes for embryo transfer (20 zygotes/female recipient) (Figs 2A and 2B). The number of rats required to produce 100 zygotes was as follows: 4, 4, 4, 5, and 8 females aged 3, 4, 5, 6, and 7 weeks, respectively. For the control, fresh zygotes derived from 3- and 7-week-old females were transferred in the same manner. The rates of development of vitrified-warmed zygotes derived from 3-, 4-, 5-, 6- and 7-week-old females into pups were 13%, 22%, 25%, 49%, and 50%, respectively. The vitrified-warmed zygotes from 6 and 7 weeks old had a higher developmental ability than those from other weeks (Table 3, Fig 2A). The incidence of development into pups was comparable between vitrified zygotes (50%) and fresh zygotes (53%) from 7-week-old females (Table 3, Fig 2C, S3 Table) (*p* = 0.6631). However, the development rate of vitrified-warmed zygotes (13%) was lower than that of fresh zygotes (34%), both from 3-week-old females (Table 3, Fig 2C, S3 Table) (*p* = 0.0105).

**Table 3:** Effect of oocyte donors’ age on the developmental rate of vitrified-warmed rat zygotes.

Age of females (weeks)	Zygotes	No. of transferred zygotes	No. of pups (%)
3	Fresh	100	34 (34.0 ± 10.8)
7	Fresh	100	53 (53.0 ± 5.7)
3	Vitrified-warmed	100	13 (13.0 ± 9.1)^a, *^
4	Vitrified-warmed	100	22 (22.0 ± 7.6)^a^
5	Vitrified-warmed	100	25 (25.0 ± 3.5)^a^
6	Vitrified-warmed	100	49 (49.0 ± 15.6)^b^
7	Vitrified-warmed	100	50 (50.0 ± 13.7)^b^

The developmental rate into pups was calculated by total no. of live pups/total no. of transferred zygotes × 100. The developmental rate into pups was compared between all vitrified-warmed groups using one-way ANOVA and Tukey’s multiple comparisons test and the different letters indicate significant differences (*p* < 0.05). The developmental rates into pups were also compared between the fresh and vitrified-warmed groups with same age using Student’s *t*-*t*est (*p < 0.05). Each recipient (*n* = 5 per experimental group) received 20 zygotes. In total, 35 recipient rats and 10 vasectomized male rats were used for embryo transfer in this experiment.

### Experiment 3: Application of vitrified-warmed zygotes for producing genetically modified rats by electroporation

Zygotes were produced via IVF using oocytes collected from 7-week-old Clr:CD females (coat color: white) and sperm collected from males of the Crl:LE strain (coat color: white with a black hood), followed by vitrification. Regarding the developmental ability, the pups of vitrified-warmed zygotes were born (developmental rate into pups = 40 ± 10%) and the coat color of all pups was white with a black hood.

**Fig 3:**
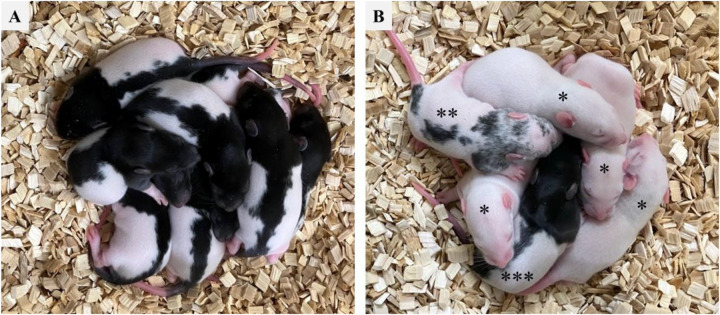
Pups derived from vitrified-warmed zygotes with or without genome editing. Pups (1-week-old) derived from vitrified-warmed zygotes produced via IVF using female Clr:CD oocytes and male Crl:LE sperm. The coat color of all pups was white with a black hood (A: without genome editing). After genome editing, we performed the embryo transfer and confirmed the coat color of the newborn pups (B: with genome editing). Excluding *albinism, we obtained **mosaic and ***black-hooded pups.

To disrupt the *Tyr* gene, we performed electroporation by introducing Cas9 ribonucleoprotein (RNP) into vitrified-warmed zygotes, particularly the morphologically normal ones (30 zygotes/cryotube × 6 = 180 zygotes). As for the control group, 60 vitrified zygotes (30 zygotes/cryotube × 2) were warmed, and 58 were then recovered. Moreover, 40 out of 54 morphologically normal zygotes were transferred to two female recipients (20 zygotes/female recipient); subsequently, 20 black-hooded pups were born (50.0%) (Table 4, Fig 3A, S2 Fig). In the genome-editing group, 170 out of 180 zygotes were recovered after warming. Additionally, 139 out of 160 morphologically normal zygotes were used for electroporation.

**Table 4:** Generation of *Tyr* mutant rats using vitrified-warmed rat zygotes.

Reagent	Pulse	No. of electroporated zygotes	No. of transferred zygotes	No. of pups (%)	No. of albinism	No. of analyzed pups	HMA + ^*^	Sequencing^**^	Mutants (%)
Tyr RNP (250 ng/μL Cas9 and 250 ng/μL gRNA)	20 V × 7	139	139	37 (26.6)	27	10	3	2	32 (86.5)
–	–	–	40	20 (50.0)	0	–	–	–	–

* The number of mutant rats identified by the heteroduplex mobility assay. ** The number of mutant rats identified by sequencing analysis. In total, 25 male and female rats were used in this experiment, and 9 recipients and 10 vasectomized male rats were used for embryo transfer.

After electroporation, all zygotes were transferred to five female rat recipients (27–31 zygotes/female recipient), resulting in 37 pups (26.6%) (Table 4). To identify *Tyr* mutants, we confirmed the coat color in each pup and then analyzed 10 pups, excluding albinism, by HMA, followed by direct sequencing analysis of PCR products. We confirmed both normal birth and mutant rates, with results comparable to those obtained in a previous report (Table 4, Fig 3B) [26].

## Discussion

This study demonstrated that 0.1 M sucrose solution for warming improved the developmental ability of vitrified-warmed rat zygotes. The vitrified-warmed zygotes derived from female rats aged 6 and 7 weeks had higher cryotolerance and developmental ability than those derived from other age categories. In addition, the vitrified-warmed zygotes can be used to produce genetically modified rats by electroporation using a genome-editing system.

In this study, we solved the technical problems of low cryotolerance and developmental ability of *in vitro*-fertilized rat zygotes. Previously, Kaneko *et al*. successfully produced genome-edited rats by using zygotes cryopreserved via the slow-freezing method. However, this method is complicated and time consuming [6]. Conversely, the vitrification method of rat zygotes is simple and quick. However, until now it had shown poor viability in vitrified-warmed zygotes [27]. Rapid warming of the vitrified zygotes and minimizing the volume of the cryopreservation solution effectively improved the viability of *in vivo*-fertilized zygotes derived from rats [28,29]. Ishizuka et al. also reported that vitrified-warmed rat zygotes via mating had higher cryotolerance and developmental ability than those via IVF [7]. *In vivo* fertilization by mating is reportedly less efficient in producing zygotes according to the rates of mating and fertilization. In our laboratory, we have efficiently produced 300 zygotes from 10 superovulated female rats by IVF (unpublished data). Our improved protocol for vitrifying and warming rat zygotes produced by IVF enhanced the efficiency of zygotes production, cryotolerance, and developmental ability in the production and preservation of genetically modified rats.

Vitrified-warmed mouse zygotes are routinely used for producing genetically engineered mice via IVF and electroporation with genome-editing technologies, attaining both high birth rates and mutation/knock-in rates [20]. However, a simple vitrification method for rat zygotes derived from IVF has not yet been established [5,9]. In the present study, we were able to increase the viability of vitrified zygotes by using a 0.1 M sucrose solution as the warming solution and oocytes collected from 6- to 7-week-old females for IVF. Furthermore, genetically modified rats were successfully produced from cryopreserved zygotes by electroporation. These results offer details of an improved protocol for the cryopreservation of rat zygotes and subsequent genome editing.

Pedro *et al*. reported that mouse zygotes cryopreserved via vitrification and warmed in various sucrose solution concentrations showed high viability when warmed in 0.5–0.75 M sucrose solution [30]. However, the percentage of morphologically normal zygotes decreased to 30% when warmed in 1.0 M sucrose solution. Generally, 0.25–0.3 M sucrose solution is used to warm vitrified rat zygotes [5,6,9,27–29]. However, in the present study, the best viability of the vitrified zygotes was obtained when warmed in a lower sucrose solution concentration of 0.1 M. The reason for this result remains unknown, but rat zygotes may be more sensitive to hypertonic stress (Table 1). This point requires further research for successful warming and recovery.

In rats, females used for IVF are generally juveniles (3–5 weeks old) because a large number of oocytes can be collected from each female [8–10,31,32]. Therefore, we previously used oocytes collected from 3- to 4-week-old females for IVF and attempted to cryopreserve the produced zygotes using the simple vitrification method. However, the rate of cryopreserved zygotes developing into pups was very low (10%) [9]. In the present study, the incidence of zygotes produced from 3- to 5-week-old female oocytes was lower than that from 6-to 7-week-old oocytes (Fig 2A). Therefore, the level of resistance to the vitrification and warming of zygotes produced via IVF derived from juvenile female rats may be lower than that from mature females. In addition, the incidence of development into pups was comparable between cryopreserved zygotes derived from 7-week-old females and fresh zygotes. However, the rate of pup development from vitrified zygotes derived from 3-week-old females was lower than that from fresh zygotes (Fig 2C). Comparing the fresh zygotes, we found that the rate of pup development from fresh zygotes derived from 3-week-old females was also lower than that from 7-week-old females (Fig 2C). Thus, zygotes from 3- to 5-week-old female oocytes may be less capable of developing into pups than those from 6- to 7-week-old female oocytes. Hirabayashi *et al.* reported that oocytes derived from rats aged 4–5 weeks were more sensitive to mechanical stress than those from >10 weeks old rats [33]. Kito reported that two-cell embryos from 3-week-old female mice had lower *in vitro* blastocyst development, nuclear counts of blastocysts, and fetal development after embryo transfer than those from 10-week-old female mice [34]. Other reports also found that oocytes from immature mice differ in several ways from those of adult mice, specifically in terms of chromosomes, ultrastructure, and epigenetics [35–37]. In rats, these key differences may be related to the lower developmental ability of zygotes from 3–5 weeks old females; although, further studies are needed to clarify this.

Genome-editing technology has been widely used to produce genetically modified rats [3], with many strains being produced to date. As mentioned above, the zygotes used for genetic modification are collected from the oviducts of females that have mated naturally with males. Thus, numerous males and females must be used to produce these zygotes, and given that rats are 10 times the size of mice (200–300 g vs. 20–30 g), an enormous breeding space is required to keep these animals. If we use superovulation and IVF techniques, several hundred zygotes can be possibly produced at once.

In this study, we showed that these zygotes could be successfully cryopreserved and that they could be warmed and genome-edited (birth rate: 26.6%, mutant: 86.5%). Honda *et al.* previously reported successful genome editing using fresh rat zygotes produced via IVF (birth rate: 28.7%, mutant: 100%) [8]. As a control, we also performed genome editing using fresh rat zygotes (birth rate: 22.1%, mutant: 95.2%; S4 Table). In the present study, genome editing using vitrified-warmed zygotes resulted in comparable or slightly lower outcomes, demonstrating the applicability of this technique to produce genetically modified rats. Enhancing the efficiency of rat zygotes production and preservation may contribute to improving animal experiments using genetically modified rats and reducing the number of animals used as oocyte donors.

In summary, using matured female rats (6–7 weeks old) as oocyte donors and 0.1 M sucrose solution for warming are suitable for the efficient cryopreservation of rat zygotes. Our findings provide an improved protocol for vitrifying and warming rat zygotes for the efficient production and preservation of genetically modified rats.

## Supporting information

S1 Fig Graphical abstract: Optimized protocol for vitrification and warming of rat zygotes using for genome editing. A 0.1-M sucrose solution for warming improved the developmental ability of vitrified-warmed zygotes (Experiment 1). The vitrified-warmed zygotes derived from female rats aged 6 and 7 weeks had higher cryotolerance and developmental ability than those derived from other age categories (Experiment 2). Moreover, the vitrified-warmed zygotes can be used to produce genetically modified rats by electroporation using a genome-editing system (Experiment 3).
(DOCX)

S2 Fig Analysis of Tyr mutant rats. Design of Tyr targeting and the results of genotyping. (A) Schematic illustration to generate mutant rat at the Tyr locus. The genomic region positioned at exon 1 was targeted by gRNA, whose target sequence is shown in black underline. Black box indicates PAM sequence. Arrows indicate the primer sets for PCR. (B) HMA results of Tyr founders. Founder numbers are shown on the upper side of pseudo-gel image. Red circles indicate HMA-positive founders. M, molecular weight markers. W, wild type. N, negative control. (C) Sequencing analysis-positive two founders. Top: the sequence chromatogram of pup #1. Bottom: the sequence chromatogram of pup #5. These two pups had both wild-type and mutated alleles.
(DOCX)

S1 Table Effects of sucrose concentrations on the survival and developmental ability of vitrified-warmed rat zygotes. (DOCX)

S2 Table Effect of oocyte donors’ age on the survival rate of vitrified-warmed rat zygotes. (DOCX)

S3 Table Effect of oocyte donors’ age on the developmental rate of vitrified-warmed rat zygotes. (DOCX)

S4 Table Generation of Tyr mutant rats using fresh rat zygotes. (DOCX)
