# Supplementary material for: Improved protocol for the vitrification and warming of rat zygotes by optimizing the warming solution and oocyte donor age
Source: PLoS One. 2025 Sep 8;20(9):e0328718. doi: 10.1371/journal.pone.0328718 (PMC12416641; doi:10.1371/journal.pone.0328718)
Supplement: S1 Fig — A 0.1-M sucrose solution for warming improved the developmental ability of vitrified-warmed zygotes (Experiment 1). The vitrified-warmed zygotes derived from female rats aged 6 and 7 weeks had higher cryotolerance and developmental ability than those derived from other age categories (Experiment 2). Moreover, the vitrified-warmed zygotes can be used to produce genetically modified rats by electroporation using a genome-editing system (Experiment 3). (DOCX) [file pone.0328718.s001.docx]

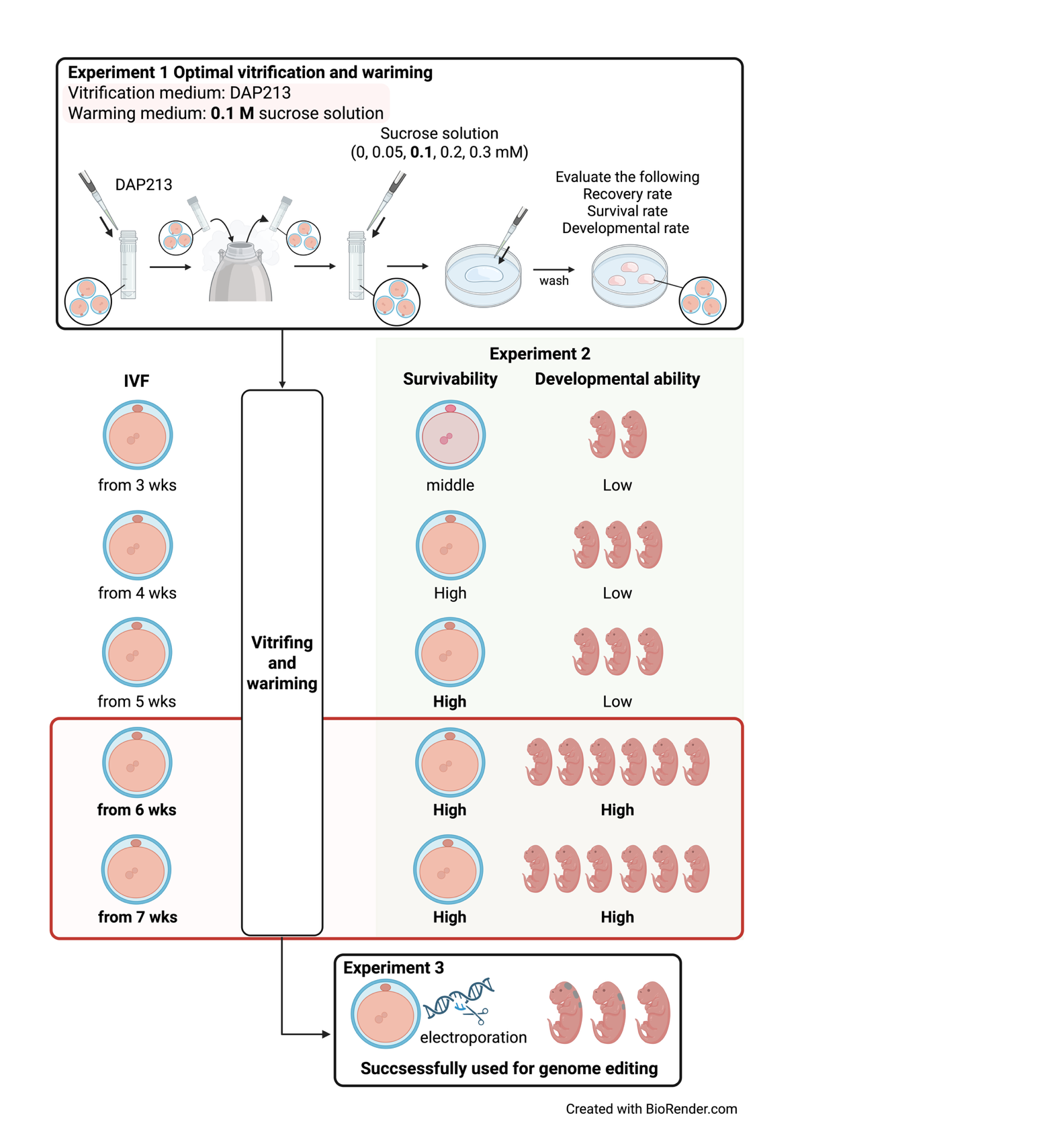


**S1 Fig. Graphical abstract: Optimized protocol for vitrification and warming of rat zygotes using for genome editing.**
