# Supplementary material for: Improved protocol for the vitrification and warming of rat zygotes by optimizing the warming solution and oocyte donor age
Source: PLoS One. 2025 Sep 8;20(9):e0328718. doi: 10.1371/journal.pone.0328718 (PMC12416641; doi:10.1371/journal.pone.0328718)
Supplement: S2 Fig — Design of Tyr targeting and the results of genotyping. (A) Schematic illustration to generate mutant rat at the Tyr locus. The genomic region positioned at exon 1 was targeted by gRNA, whose target sequence is shown in black underline. Black box indicates PAM sequence. Arrows indicate the primer sets for PCR. (B) HMA results of Tyr founders. Founder numbers are shown on the upper side of pseudo-gel image. Red circles indicate HMA-positive founders. M, molecular weight markers. W, wild type. N, negative control. (C) Sequencing analysis-positive two founders. Top: the sequence chromatogram of pup #1. Bottom: the sequence chromatogram of pup #5. These two pups had both wild-type and mutated alleles. (DOCX) [file pone.0328718.s002.docx]

**S2 Fig. Analysis of Tyr mutant rats.**
