# Supplementary material for: Improved protocol for the vitrification and warming of rat zygotes by optimizing the warming solution and oocyte donor age
Source: PLoS One. 2025 Sep 8;20(9):e0328718. doi: 10.1371/journal.pone.0328718 (PMC12416641; doi:10.1371/journal.pone.0328718)
Supplement: S1 Table — (DOCX) [file pone.0328718.s003.docx]

**S1 Table Effects of sucrose concentrations on the survival and developmental ability of vitrified-warmed rat zygotes**

| **Sucrose**  **(M)** | **No. of**  **vitrified zygotes** | **No. of recovered zygotes** | **%** | **No. of survived zygotes** | **%** | **No. of two-cell**  **embryos** | **%** |
| --- | --- | --- | --- | --- | --- | --- | --- |
| 0 | 20  20  20  20  20 | 20  19  19  19  20 | 100.0  95.0  95.0  95.0  100.0 | 15  13  6  10  19 | 75.0  68.4  31.6  52.6  95.0 | 9  11  4  6  12 | 45.0  57.9  21.1  31.6  60.0 |
|  | **100** | **97** | **97.0** | **63** | **64.9** | **42** | **43.3** |
| 0.05 | 20  20  20  20  20 | 20  19  20  19  20 | 100.0  95.0  100.0  95.0  100.0 | 14  18  17  17  7 | 70.0  94.7  85.0  89.5  35.0 | 11  12  14  14  4 | 55.0  63.2  70.0  73.7  20.0 |
|  | **100** | **98** | **98.0** | **73** | **74.5** | **55** | **56.1** |
| 0.1 | 20  20  20  20  20 | 20  20  19  20  19 | 100.0  100.0  95.0  100.0  95.0 | 20  18  17  17  19 | 100.0  90.0  89.5  85.0  100.0 | 13  16  16  14  19 | 65.0  80.0  84.2  70.0  100.0 |
|  | **100** | **98** | **98.0** | **91** | **92.9** | **78** | **79.6** |
| 0.2 | 20  20  20  20  20 | 18  20  19  18  20 | 90.0  100.0  95.0  90.0  100.0 | 16  19  15  9  10 | 88.9  95.0  78.9  50.0  50.0 | 11  16  12  3  9 | 61.1  80.0  63.2  16.7  45.0 |
|  | **100** | **95** | **95.0** | **69** | **72.6** | **51** | **53.7** |
| 0.3 | 20  20  20  20  20 | 19  19  20  20  20 | 95.0  95.0  100.0  100.0  100.0 | 17  11  10  14  13 | 89.5  57.9  50.0  70.0  65.0 | 9  7  7  8  6 | 47.4  36.8  35.0  40.0  30.0 |
|  | **100** | **98** | **98.0** | **65** | **66.3** | **37** | **37.8** |
