# Supplementary material for: Improved protocol for the vitrification and warming of rat zygotes by optimizing the warming solution and oocyte donor age
Source: PLoS One. 2025 Sep 8;20(9):e0328718. doi: 10.1371/journal.pone.0328718 (PMC12416641; doi:10.1371/journal.pone.0328718)
Supplement: S2 Table — (DOCX) [file pone.0328718.s004.docx]

**S2 Table Effect of oocyte donors’ age on the survival rate of vitrified-warmed rat zygotes**

| **Age of females**  **(weeks)** | **No. of**  **vitrified zygotes** | **No. of recovered zygotes** | **%** | **No. of survived zygotes** | **%** |
| --- | --- | --- | --- | --- | --- |
| 3 | 30  30  30  30  30 | 28  29  27  28  28 | 93.3  96.7  90.0  93.3  93.3 | 23  22  23  21  21 | 82.1  75.9  85.2  75.0  75.0 |
|  | **150** | **140** | **93.3** | **110** | **78.6** |
| 4 | 30  30  30  30  30 | 28  28  27  28  28 | 93.3  93.3  90.0  93.3  93.3 | 26  25  25  27  26 | 92.9  89.3  92.6  96.4  92.9 |
|  | **150** | **139** | **92.7** | **129** | **92.8** |
| 5 | 30  30  30  30  30 | 28  30  27  27  28 | 93.3  100.0  90.0  90.0  93.3 | 28  28  26  25  23 | 100.0  93.3  96.3  92.6  82.1 |
|  | **150** | **140** | **93.3** | **130** | **92.9** |
| 6 | 30  30  30  30  30 | 28  30  29  29  30 | 93.3  100.0  96.7  96.7  100.0 | 27  28  28  27  29 | 96.4  93.3  96.6  93.1  96.7 |
|  | **150** | **146** | **97.3** | **139** | **95.2** |
| 7 | 30  30  30  30  30 | 28  30  30  30  30 | 93.3  100.0  100.0  100.0  100.0 | 28  28  28  30  30 | 100.0  93.3  93.3  100.0  100.0 |
|  | **150** | **148** | **98.7** | **144** | **97.3** |
