# Supplementary material for: Improved protocol for the vitrification and warming of rat zygotes by optimizing the warming solution and oocyte donor age
Source: PLoS One. 2025 Sep 8;20(9):e0328718. doi: 10.1371/journal.pone.0328718 (PMC12416641; doi:10.1371/journal.pone.0328718)
Supplement: S3 Table — (DOCX) [file pone.0328718.s005.docx]

**S3 Table Effect of oocyte donors’ age on the developmental rate of vitrified-warmed rat zygotes**

| **Age of females**  **(weeks)** | **Zygotes** | **No. of transferred zygotes** | **No. of pups** | **%** |
| --- | --- | --- | --- | --- |
| 3 | Fresh | 20  20  20  20  20 | 5  8  5  6  10 | 25.0  40.0  25.0  30.0  50.0 |
|  |  | **100** | **34** | **34.0** |
| 7 | Fresh | 20  20  20  20  20 | 9  11  10  11  12 | 45.0  55.0  50.0  55.0  60.0 |
|  |  | **100** | **53** | **53.0** |
| 3 | Vitrified-warmed | 20  20  20  20  20 | 4  1  2  1  5 | 20.0  5.0  10.0  5.0  25.0 |
|  |  | **100** | **13** | **13.0** |
| 4 | Vitrified-warmed | 20  20  20  20  20 | 2  5  5  4  6 | 10.0  25.0  25.0  20.0  30.0 |
|  |  | **100** | **22** | **22.0** |
| 5 | Vitrified-warmed | 20  20  20  20  20 | 4  5  5  5  6 | 20.0  25.0  25.0  25.0  30.0 |
|  |  | **100** | **25** | **25.0** |
| 6 | Vitrified-warmed | 20  20  20  20  20 | 7  11  6  13  12 | 35.0  55.0  30.0  65.0  60.0 |
|  |  | **100** | **49** | **49.0** |
| 7 | Vitrified-warmed | 20  20  20  20  20 | 6  12  10  9  13 | 30.0  60.0  50.0  45.0  65.0 |
|  |  | **100** | **50** | **50.0** |
