# Supplementary material for: Improved protocol for the vitrification and warming of rat zygotes by optimizing the warming solution and oocyte donor age
Source: PLoS One. 2025 Sep 8;20(9):e0328718. doi: 10.1371/journal.pone.0328718 (PMC12416641; doi:10.1371/journal.pone.0328718)
Supplement: S4 Table — (DOCX) [file pone.0328718.s006.docx]

**S4 Table Generation of *Tyr* mutant rats using fresh rat zygotes**

| Reagent | Pulse | No. of electroporated zygotes | No. of transferred zygotes | No. of pups  (%) | No. of albinism | No. of analyzed pups | Sequencing^*^ | Mutants  (%) |
| --- | --- | --- | --- | --- | --- | --- | --- | --- |
| Tyr RNP  (250 ng/μL Cas9 and 250 ng/μL gRNA) | 20 V  ×  7 | 116 | 95 | 21  (22.1) | 20 | 1 | 0 | 20  (95.2) |
| - | - | - | 40 | 20 (50.0) | 0 | - | - | - |

^*^The number of mutant rats identified by sequencing analysis.
